# Endothelial cell injury by high glucose and heparanase is prevented by insulin, heparin and basic fibroblast growth factor

**DOI:** 10.1186/1475-2840-4-12

**Published:** 2005-08-09

**Authors:** Juying Han, Anil K Mandal, Linda M Hiebert

**Affiliations:** 1 Department of Veterinary Biomedical Sciences, University of Saskatchewan, Saskatoon, Saskatchewan, S7N 5B4, Canada; 2 Department of Medicine, University of Florida, Jacksonville, Florida, 32086, USA

## Abstract

**Background:**

Uncontrolled hyperglycemia is the main risk factor in the development of diabetic vascular complications. The endothelial cells are the first cells targeted by hyperglycemia. The mechanism of endothelial injury by high glucose is still poorly understood. Heparanase production, induced by hyperglycemia, and subsequent degradation of heparan sulfate may contribute to endothelial injury. Little is known about endothelial injury by heparanase and possible means of preventing this injury.

**Objectives:**

To determine if high glucose as well as heparanase cause endothelial cell injury and if insulin, heparin and bFGF protect cells from this injury.

**Methods:**

Cultured porcine aortic endothelial cells were treated with high glucose (30 mM) and/or insulin (1 U/ml) and/or heparin (0.5 *μ*g/ml) and /or basic fibroblast growth factor (bFGF) (1 ng/ml) for seven days. Cells were also treated with heparinase I (0.3 U/ml, the *in vitro *surrogate heparanase), plus insulin, heparin and bFGF for two days in serum free medium. Endothelial cell injury was evaluated by determining the number of live cells per culture and lactate dehydrogenase (LDH) release into medium expressed as percentage of control.

**Results:**

A significant decrease in live cell number and increase in LDH release was found in endothelial cells treated with high glucose or heparinase I. Insulin and/or heparin and/or bFGF prevented these changes and thus protected cells from injury by high glucose or heparinase I. The protective ability of heparin and bFGF alone or in combination was more evident in cells damaged with heparinase I than high glucose.

**Conclusion:**

Endothelial cells injured by high glucose or heparinase I are protected by a combination of insulin, heparin and bFGF, although protection by heparin and/or bFGF was variable.

## Background

Diabetes mellitus is characterized by hyperglycemia and vascular complications including microangiopathy and macroangiopathy [1,2]. The hallmarks of diabetic microangiopathy are retinopathy and nephropathy leading to blindness and renal failure respectively [3,4]. Macroangiopathy in diabetes, includes coronary artery disease, peripheral vascular disease, and cerebrovascular disease, and results from an acceleration of atherosclerosis and increased thrombosis thus increasing the risk of myocardial infarction, stroke and ischaemia [5,6]. It is reported that under better glycemic control fewer patients develop eye and/or renal complications [7].

Since the initial injury by hyperglycemia occurs in the blood vessel, endothelial cells (ECs) are considered to be the first target. Heparan sulfate proteoglycans (HSPGs), an important EC component, are synthesized by ECs and incorporated into the plasma membrane and extracellular matrix (ECM) [8,9]. In the ECM, HSPGs interact with fibronectin, laminin, collagen and growth factors such as basic fibroblast growth factor (bFGF) and help maintain vascular integrity [10,11]. HSPGs with their negative charged sulfate and carboxylate residues, create a "charge barrier", which decrease the permeability of anionic plasma proteins [12]. Thus degradation of HSPGs could lead to an increase in vascular permeability, decrease in vascular integrity, and changes in growth factor activity. Depletion of heparan sulfate (HS) and/or abnormal glycosaminoglycan (GAG) metabolism appears to be a pivotal mechanism associated with diabetic EC injury. HSPG or HS were decreased in the glomerular basement membrane (GBM) of patients with overt diabetic nephropathy which correlated with the degree of proteinuria [13,14]. A similar decrease in HS content was observed in the aortic intima of diabetic patients [15]. Skin basement membrane thickness was significantly reduced in patients with diabetic nephropathy compared to those without nephropathy. As well, HSPG synthesis was decreased in aorta, liver and intestinal epithelium of diabetic rats [16-18]. Thus in the diabetic condition, changes in HS metabolism may occur in any tissue suggesting the link between HS abnormalities and vascular complications in both large and small vessels.

Heparanase is an endo-*β*-D-glucuronidase that cleaves HS at specific interchain sites. Under normal physiological conditions, heparanase is expressed in platelets, cytotrophoblasts, mast cells, neutrophils, macrophages, and the placenta [19]. Heparanase activity was found in the urine of some diabetic patients and heparanase protein was expressed in both the glomerular mesangial and epithelial cell lysates, but not in intact cells [20]. HSPG degradation by heparanase upregulation may contribute to EC injury by hyperglycemia. Thus we wished to determine if heparanase as well as high glucose injured ECs.

Insulin and heparin alone, or in combination, prevented the intercellular gaps formed in ECs cultured in high glucose [21]. Several previous studies have shown that insulin increases nitric oxide (NO) production in cultured ECs and ensures normal vascular function [22,23]. Heparin can accumulate in ECs at a greater concentration than in plasma, increase HS on the EC surface, and prevent ECs from free radical injury [24-26]. Therefore, we postulated that insulin and/or heparin would protect ECs from high glucose or heparanase injury.

bFGF has a high affinity for heparin and HS which is required for interaction with its receptor (bFGFR). However, HS in the ECM also limits bFGF release into interstitial spaces [27,28]. The high affinity of bFGF for heparin and HS, together with the EC proliferation potential of bFGF [29], may protect ECs. Previous studies have shown that both heparanase and plasmin degrade HSPG and decrease the stability of the bFGF/HS/bFGFR complex resulting in loss of bFGF which was corrected by exogenous heparin [30,31]. Thus, the stabilization of bFGF/HS/bFGFR complex, by supplying heparin and bFGF, may protect ECs from injury by high glucose or heparanase.

Therefore, the purposes of our study were to determine if high glucose or heparanase induced cultured EC injury and if supplementation of insulin, heparin and bFGF would protect ECs from this damage.

## Methods

### Culture of Porcine Aortic Endothelial Cells (PAECs)

PAECs were cultured according to the method of Gotlieb and Spector [32]. Porcine aortic segments were rinsed in three changes of calcium- and magnesium-free Dulbecco's phosphate-buffered saline (CMF-DPBS), while the adventitial connective tissue and end pieces were trimmed. One end of the aorta was clamped with two hemostats (ensuring no leakage from bottom or branch points) and the lumen was rinsed three times with CMF-DPBS and then filled with collagenase solution (Type IV, SIGMA, St. Louis, MO, USA; 1 mg/ml in CMF-DPBS at 37°C). After 6 minutes the collagenase solution was removed and the endothelial surface was gently rinsed with M199 (GibcoBRL, Life Technologies, Inc., Grand Island, NY, USA) containing 5% fetal bovine serum (FBS, GibcoBRL), 50 *μ*g/ml penicillin (SIGMA) and 10 *μ*g/ml streptomycin (SIGMA). The medium was removed and plated onto 60 mm culture dishes. The volume was made up to 2 ml medium/dish. Cells were incubated at 37°C with 5% CO_2 _/ 95% air in a humidified environment. ECs were identified by their morphological appearance of cobblestone-like flattened cells and the presence of von Willebrand Factor (vWF) in initial cultures. Non-endothelial-like cells, such as smooth muscle cells and fibroblasts, were destroyed by mechanical suction before the first passage.

To subculture, confluent 60 mm dishes of PAECs were washed twice with sterile CMF-DPBS, followed by exposure to a sterile 0.025% trypsin solution for two or three minutes at room temperature. The cells were then resuspended in 6 ml of culture medium and seeded onto three 60 mm dishes (2 ml/dish). Confluent cultures at passage 4, in 35 mm dishes, were used in experiments.

### Reagents

Reagents were first prepared as stock solutions in CMF-DPBS at the following concentrations: glucose (D-Glucose, BDH Inc. Toronto, Canada) 3M, unfractionated bovine lung heparin (151 USP U/mg Upjohn Pharmaceuticals, Kalamazoo, MI, USA) 0.1 mg/ml, insulin (Humulin^® ^N) 100 U/ml. Stock solutions of heparinase I (SIGMA) 10 and 1 U/ml and bFGF (SIGMA) 0.1 ng/*μ*l were prepared in M199 without serum.

### Cell Treatment

Cell medium was changed just prior to addition of reagents to 1 ml of medium per dish. For high glucose, 10 *μ*l of 3 M stock solution was added to give a final added concentration of 30 mM. For heparin, 5 *μ*l of 0.1 mg/ml of stock solution was added to give a final concentration 0.5 *μ*g/ml. For insulin, 10 *μ*l of 100 U/ml stock solution was added to give a final concentration of 1 U/ml. For bFGF, 10 *μ*l of 0.1 ng/*μ*l stock solution was added to give a final concentration of 1 ng/ml. Cell medium was changed and reagents were added fresh every other day for seven days. Cells were harvested 48 hours after the last addition.

In order to determine the culture conditions and damaging doses of heparinase I in PAECs, heparinase I was added to cultures at concentrations of 0.01, 0.05, 0.1, 0.3 and 0.5 U/ml in medium, which was produced by adding 10 *μ*l of 1 U/ml and 5, 10, 30 and 50 *μ*l of 10 U/ml of heparinase I to 1 ml medium respectively. Cells were cultured either in medium with serum for six or ten days, where cell medium was changed and fresh heparinase I was added every other day; or in serum free medium for two days by adding heparinase I once.

To determine the effect of heparin, insulin and bFGF in the presence heparinase I, cell medium was changed to M199 without serum. Then 30 *μ*l of 10 U/ml of heparinase I was added to give a final concentration of 0.3 U/ml. Then heparin, insulin or bFGF was added to medium as described above. Cells were harvested two days later.

### Assessment of Cell Injury

#### Trypan blue exclusion

Number of viable cells was determined by using trypan blue dye. The cells were washed with CMF-DPBS, detached from the culture dishes using 0.5 ml of 0.025% trypsin for 2–3 minutes and then were suspended in 1 ml of culture medium. An aliquot of 100 *μ*l of the cell suspension was mixed with 10 *μ*l of 0.4% of trypan blue solution (SIGMA) for 2–3 minutes. Cells were counted in a hemocytometer chamber with the light microscope. The number of live cells, those excluding trypan blue, and dead cells, those taking up trypan blue, were counted and calculated per dish. The number of live cells in experimental cultures was expressed as percent of live cells in control cultures.

#### Lactate dehydrogenase (LDH) assay

Cell medium (100 *μ*l) from each culture was saved in a microcentrifuge tube for LDH determination using Sigma Diagnostic Kit No. 228-UV. A 50 *μ*l sample was added to 500 *μ*l of reagent, and LDH was quantified spectrophotometrically by the rate of change in absorbance at 340 nm at room temperature. The increased absorbance at 340 nm is the result of reduction of NAD^+ ^to NADH as LDH catalyzes the conversion of lactate to pyruvate, thus the rate of NADH production is directly proportional to the LDH activity in the sample.

### Statistical Analysis

All data was expressed as mean +/- standard error (SE) from three dishes/group. A one-way ANOVA was used to determine significant differences between groups. Values of *P *< 0.05 were considered to be statistically significant.

## Results

### Effect of Heparin and Insulin on PAECs Injured by High Glucose

**Figure 1:**
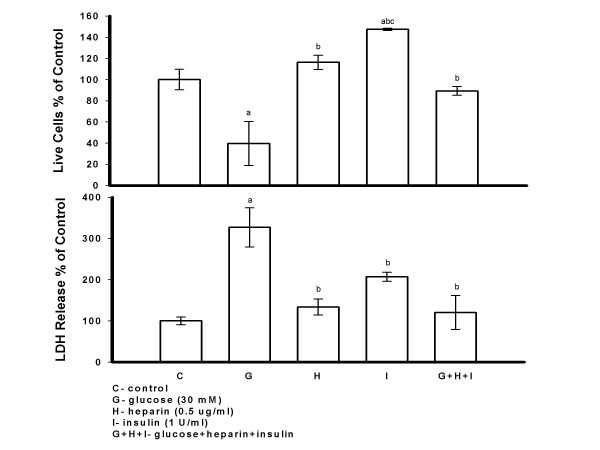
**PAECs Injured by High Glucose were Protected by a Combination of Heparin and Insulin**. PAECs were exposed to glucose (30 mM), insulin (1 U/ml), heparin (0.5 *μ*g/ml) and glucose plus heparin plus insulin for seven days. Cell medium was changed and fresh reagents were added every other day. Cells were counted and LDH release in medium was determined 48 hrs after the last addition of reagents. Results are expressed as mean +/- SE of three dishes per group. Significantly different than a, control; b, glucose; c, glucose+heparin+insulin (*P *< 0.01) (one-way ANOVA).

PAECs were exposed to high glucose (30 mM) alone, insulin (1 U/ml) alone, heparin (0.5 *μ*g/ml) alone and glucose plus heparin plus insulin for seven days. PAECs treated with high glucose showed a significant decrease in live cell number and increase in LDH release compared to control cells. Compared to control cells, there were significant changes in live cell number and LDH release in insulin alone treated cells, but not in heparin alone treated cells. Live cell number was significantly greater in heparin or insulin alone versus high glucose treated cultures. The combination of heparin and insulin in the presence of high glucose significantly increased live cell number and decreased LDH release compared to cells injured by high glucose alone (Figure 1).

**Figure 2:**
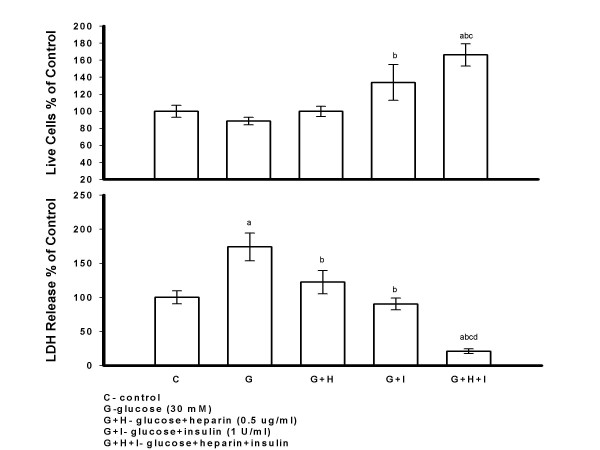
**The Protective Effect of Insulin and/or Heparin on PAECs Injured by High Glucose**. PAECs were treated with glucose (30 mM), glucose plus insulin (1 U/ml), glucose plus heparin (0.5 *μ*g/ml) and glucose plus insulin plus heparin for seven days. Cell medium was changed and fresh reagents were added every other day. Cells were counted and LDH release to medium was determined 48 hrs after the last addition of reagents. Results are expressed as mean +/- SE of three dishes per group. Significantly different than a, control; b, glucose; c, glucose + heparin; d, glucose + insulin (*P *< 0.01) (one-way ANOVA).

To determine if insulin and /or heparin protect PAECs from high glucose injury, PAECs were treated with high glucose (30 mM), glucose plus insulin (1 U/ml), glucose plus heparin (0.5 *μ*g/ml), and glucose plus insulin plus heparin for seven days (Figure 2). A trend towards a decrease in live cell number and a significant increase in LDH release were seen in PAECs treated with high glucose compared to control cultures. A significant increase in live cell number and decrease in LDH release was seen in PAECs treated with high glucose and a combination of insulin and heparin compared to high glucose treatment alone, similar to results shown in Figure 1. A significant increase in live cell number and decrease in LDH release was seen when insulin was added to high glucose injured cells. High glucose plus heparin treated cultures showed a trend towards an increase in live cell number, and a significant decrease in LDH release compared to high glucose treatment alone (Figure 2).

### Effect of Insulin and/or Heparin on PAECs Injured by High Glucose in the Presence of bFGF

**Figure 3:**
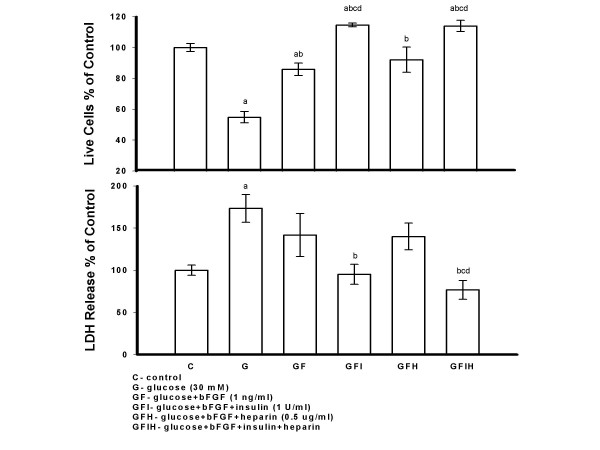
**Insulin and/or Heparin Protected PAECs from High Glucose Injury when bFGF was Present in Cell Medium**. PAECs were treated with glucose (30 mM), glucose plus bFGF (1 ng/ml), glucose plus bFGF plus insulin (1 U/ml), glucose plus bFGF plus heparin (0.5 *μ*g/ml) and glucose plus bFGF plus insulin plus heparin for seven days. Cell medium was changed and fresh reagents were added every other day. Cells were counted and medium LDH was determined 48 hrs after the last addition of reagents. Results are expressed as mean +/- SE of three dishes per group. Significantly different than a, control; b, glucose; c, glucose+bFGF; d, glucose+bFGF+heparin (*P *< 0.05) (one-way ANOVA).

PAECs were treated with high glucose (30 mM), glucose plus bFGF (1 ng/ml), glucose plus bFGF plus insulin (1 U/ml), glucose plus bFGF plus heparin (0.5 *μ*g/ml) and glucose plus bFGF plus insulin plus heparin for seven days. A significant decrease in live cell number and increase in LDH release was shown in high glucose treated versus control cells. When bFGF was present in cell medium, the combination of insulin and heparin had a protective effect on high glucose injured cells as shown by a significant increase in live cell number and decrease in LDH release in cells treated with glucose plus bFGF plus insulin plus heparin versus high glucose alone. In addition, a significant increase in live cell number and decrease in LDH release was shown in cells treated with high glucose plus insulin plus bFGF versus glucose alone. Heparin with bFGF or bFGF added to high glucose treated cells showed a significant increase in live cell number versus high glucose treatment alone. Although LDH release was less in high glucose plus heparin plus bFGF and high glucose plus bFGF versus the high glucose alone treated cells, this difference did not reach significance. The combination of insulin plus bFGF, and insulin plus heparin plus bFGF was more protective than bFGF or bFGF plus heparin on high glucose treated cultures, when numbers of live cells were considered. The combination of insulin plus heparin plus bFGF was more protective than bFGF plus heparin when LDH was considered (Figure 3).

### Damaging Effect of Heparinase I on PAECs

PAECs were exposed to different doses of heparinase I (0.01, 0.05, 0.1 and 0.3 U/ml) for six or ten days in M199 with 5% serum. Cells were harvested 24 hours after the last addition of heparinase I. There were no significant differences in live cell number and LDH release in control cultures compared to those treated with different doses of heparinase I. PAECs exposed to heparinase I (0.05, 0.1, 0.3 and 0.5 U/ml) for 48 hours in serum free M199 showed a significant decrease in cell viability and increase in LDH release compared to the control group. Cell injury was dose dependent since there was a significant decrease in cell viability, with heparinase I 0.5 U/ml compared to 0.05 U/ml (data not shown). Doses of 0.3 U/ml heparinase I in serum free media conditions were chosen for the following experiments.

### Effect of Insulin, Heparin and bFGF on Heparinase I Induced PAEC Injury

**Figure 4:**
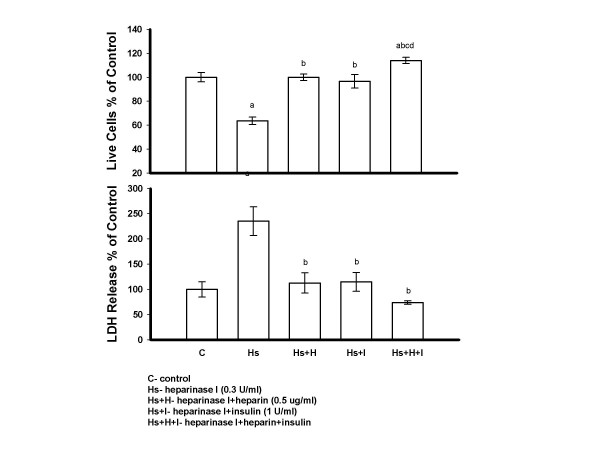
**Heparinase I Induced PAECs Injury was Prevented by Insulin and/or Heparin**. PAECs were treated with heparinase I (0.3 U/ml) and/or insulin (1 U/ml) and/or heparin (0.5 *μ*g/ml) for 48 hrs in serum free medium 199, then cells were counted and media LDH was determined. Insulin and/or heparin were added immediately after heparinase I addition. All reagents were only added once. Results are expressed as mean +/- SE of three dishes per group. Significantly different than a, control; b, heparinase I; c, heparinase I+heparin; d, heparinase I+insulin (*P *< 0.005) (one-way ANOVA).

PAECs were treated with heparinase I (0.3 U/ml) and/or insulin (1 U/ml) and/or heparin (0.5 *μ*g/ml) for 48 hours in serum free M199. Treatment with heparinase I showed a significant decrease in live cell number and increase in LDH release compared to control cells. Addition of insulin or heparin to heparinase I treated cells showed a significant increase in live cell number and decrease in LDH release compared to heparinase I treatment alone. Furthermore, the combination of insulin and heparin showed a significant increase in live cell number compared to all other groups, the LDH levels were also the lowest in this group (Figure 4).

**Figure 5:**
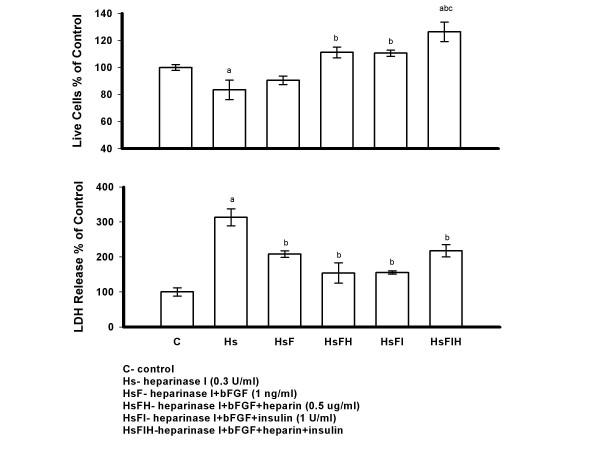
**The Protective Effect of Insulin and/or Heparin on PAECs Injured by Heparinase I when bFGF was Present in Cell Medium**. PAECs were treated with heparinase I (0.3 U/ml), heparinase I plus bFGF (1 ng/ml), heparinase I plus insulin (1 U/ml) plus bFGF, heparinase I plus heparin (0.5 *μ*g/ml) plus bFGF and heparinase I plus insulin plus heparin plus bFGF for 48 hrs in serum free medium 199. After 48 hrs, cells were counted and media LDH was determined. Insulin, heparin and bFGF were added immediately after heparinase I addition. Results are expressed as mean +/- SE of three culture dishes per group. Significantly different than a, control ; b, heparinase I; c, heparinase I+bFGF (*P *< 0.05) (one-way ANOVA).

To determine the protective effect of insulin and/or heparin on PAECs injured by heparinase I when bFGF was present in cell medium, PAECs were treated with heparinase I, heparinase I plus bFGF (1 ng/ml), heparinase I plus insulin plus bFGF, heparinase I plus heparin plus bFGF and heparinase I plus insulin plus heparin plus bFGF for 48 hours in serum free M199. Heparinase I treated PAECs showed a significant decrease in live cell number and increase in LDH release compared control cells. Cells treated with heparinase I and bFGF showed a significant decrease in LDH release, but not an increase in live cell number when compared to heparinase I treated cells. A significant increase in live cell number and decrease in LDH release was seen in cultures treated with bFGF plus insulin, bFGF plus heparin and bFGF plus insulin plus heparin in the presence of heparinase I versus heparinase I treatment alone. Furthermore, when compared to bFGF, bFGF plus insulin plus heparin showed a significant increase in live cell number in the presence of heparinase I (Figure 5).

## Discussion

Vascular complications are the main causes of morbidity and mortality in diabetes mellitus. ECs play a pivotal role in the regulation of vascular tone, as well as in the maintenance of vascular integrity, blood fluidity and homeostasis. EC injury is the initial step leading to irreversible structural abnormalities, followed by progressive microvascular occlusion in the eye and kidney as well as intimal proliferation in large vessels [33-35]. The exact cause of EC injury is still unclear.

In this study, PAECs were used as an *in vitro *model to study human vascular disease associated with EC injury since there is a similarity between human and porcine tissue [36]. ECs of both micro and macro vascular origin present similar pathological features in diabetic complications. Thus ECs from porcine aorta (macro vessel) injured by high glucose could mimic EC injury associated with uncontrolled hyperglycemia in diabetes. High glucose injury in our model agrees with previous observations of ECs grown under hyperglycemic conditions showing decreased proliferation and fibrinolytic potential and increased programmed cell death [37,38]. It was previously reported that normal human umbilical vein ECs showed increased proliferation when cultured in medium with high glucose (30 mM) for ten to twelve days [39], a longer period compared to the seven day treatment in our study. Cell proliferation increased similarly when umbilical ECs were obtained from pregnant diabetic women [40]. PAECs treated with high glucose for seven days in the present study may represent early forms of injury and showed reduced live cell number indicating decreased cell proliferation. Some variation exists in the response of ECs to high glucose conditions. Cell conditions such as variation between animals, subtle differences in medium, CO_2 _levels, humidity, and other unidentified factors may be responsible for this variation.

Depletion and abnormalities in HS and HSPG have been found in the kidney, skin and aortic intima of diabetic patients with nephropathy [13-15,41]. The degradation of HSPG may play a role in EC injury leading to diabetic vascular complications. Heparanase cleaves HS chains at specific sites and may be responsible for HSPG degradation contributing to EC injury. Heparanase has been found in the kidney and urine of diabetic patients [20]. In order to determine if heparanase as well as high glucose damage ECs, PAECs were treated with heparinase I.

Several heparanases have been purified and characterized from platelets, placenta, and Chinese Hamster Ovary (CHO) cells including connective tissue activating peptide III (CTAP-III), Hpa I, Hpa II and CHO cell heparanases [42]. Heparinase I, from *Flacobacterium heparinum (Cytophagia heparinia)*, the commercially available heparanase, was chosen for PAEC treatment, and cleaves HS [43]. Heparinase I did not cause injury when PAECs were cultured in M199 with serum for six to ten days, but showed a dose effect when cultured in serum-free medium for two days. These findings suggest that a serum constituent inhibits heparanase activity. A recently discovered cell surface protein, HS/heparin-interacting protein (HIP), was shown to prevent heparanase access to its substrate HS by competing with the same binding recognition site as in the HS chain [44,45]. Thus in our experiments, serum may contain HIP so that heparanase was active only in serum free medium.

Our findings showing cell injury with both high glucose and heparinase I treatment suggest that high glucose may induce heparanase upregulation which degrades HS causing cell injury. This injury occurs in the presence of serum which would contain HIP that may interact with heparanase at the cell surface. This suggests that with high glucose, heparanase may be produced within the cell. Heparanase activity is optimal between pH 5.0 and 6.5, with much less activity above pH 7.0 [46]. Glucose (30 mM) added for seven days to ECs lowers the medium pH (medium color become yellow) and may further stimulate heparanase activity.

Exogenous heparin significantly reduces proteinuria in diabetic patients and animals [47,48]. Heparin promotes antioxidant and barrier properties of blood vessels, prevents the formation of occlusive vascular thrombi, protects against proteolytic or oxidative damage, and lowers blood pressure [26,49,50]. Heparin and HS, similar in chemical structure, possess common physiological and biological features important in the vasculature. Heparin modifies the synthesis and the structure of HSPG [51,52]. In our study, addition of heparin to heparinase I treated ECs significantly increased live cell number and decreased LDH release compared to ECs treated with heparinase I alone suggesting that heparin has the ability to prevent cell injury by heparanase. A significant decrease in LDH release and a trend towards an increase in live cell number (close to control levels) seen in high glucose and heparin treated cells compared to high glucose alone also indicate the potential of heparin to protect ECs injured by high glucose.

HS and heparin have high affinity for bFGF and are part of the bFGF/bFGFR complex that affects the growth, differentiation and migration of many cell types [53]. Thus, bFGF function is protected by HS synthesis and perturbed by its degradation. Our results showed a significant increase in live cell number and a trend towards a decrease in LDH release both in cells treated with high glucose plus bFGF and high glucose plus bFGF plus heparin when compared to high glucose alone indicating some protective effects of bFGF. However, the live cell number in controls is significantly greater than high glucose plus bFGF indicating bFGF alone dose not eliminate high glucose injury. Since high glucose produces many metabolic and biochemical abnormalities through several cellular pathways, normal bFGF function may be altered by its interaction with abnormal metabolites. Previous studies showed that in hyperglycemia, nonenzymatic glycosylation of bFGF decreased bFGF activity [54] and could explain our observations here. Moreover, we observed similar results when ECs were damaged by heparinase I. The protective effect of bFGF on heparinase I injury is shown by a significantly decreased LDH release and a trend towards an increase in cell number compared to heparinase I injury alone. This protective ability of bFGF is consistent with that seen in high glucose plus bFGF treated ECs.

The binding of heparin to bFGF depends on the molecular mass, degree of sulfation and the disaccharide composition. Unfractionated bovine lung heparin used here is highly sulfated and of high molecular weight and has previously been shown to protect bFGF from tryptic cleavage. This capacity was reduced by N-desulfation and N-acetylation of the bovine lung heparin [55]. Previous studies have also suggested that heparin first needs to bind to the cell surface to fulfill the role of heparan sulfate in bFGF receptor interactions [56]. We have observed that bovine lung heparin binds to the surface of cultured porcine endothelial cells and thus would be able to interact with bFGF [57].

When heparin was added to ECs treated with heparinase I and bFGF, live cell number increased and LDH release decreased significantly compared to heparinase I treatment alone and showed a more pronounced increase in live cells and decrease in LDH than addition of bFGF alone suggesting that bFGF and heparin bind together to prevent HSPG from degradation by heparinase I. These findings cause us to speculate that heparin may exert its protective effect in two steps: firstly, heparin increases EC synthesis of HS; secondly, newly synthesized HS with exogenous bFGF and heparin form the bFGF/HS or heparin/ bFGFR complex which allows bFGF to play its physiological role in cell growth, differentiation, proliferation. As well, in the case of heparanase injury, heparin in the medium may compete with HS for heparanase and thus may prevent the degradation of HS.

Insulin not only stimulates cells to utilize glucose, but also promotes DNA synthesis and cell growth. The latter effect was supported in this study when ECs, treated with insulin alone, significantly increased live cell number compared to controls (Figure 1). Insulin protection of high glucose or heparinase I treated ECs was shown in all treatment combinations including insulin alone, insulin plus heparin, insulin plus bFGF and insulin plus heparin plus bFGF. The mechanism by which insulin protects ECs from high glucose injury is not entirely understood. Other vasoprotective actions of insulin are its ability to increase NO production [58], act as an antioxidant and prevent atherosclerosis by reducing oxygen consumption [59]. Our present study suggests that the combination of insulin, heparin, and bFGF may have additive effects with significantly increased live cell number and decreased LDH compared to high glucose or heparinase I alone. With high glucose injury the three combined treatments were more effective than bFGF and bFGF plus heparin and suggested increased effectiveness compared to insulin plus bFGF when medium LDH levels were considered. With heparinase I injury combined treatments were significantly more effective than bFGF with a tendency towards increased effectiveness with heparin or insulin plus bFGF when live cell number was considered.

## Conclusion

This study demonstrates that both high glucose and heparinase I cause EC injury and suggests a link between hyperglycemia and heparanase induction in diabetic complications. Exogenous heparinase I damages ECs only in serum free conditions. The mechanism of EC injury by high glucose is a complicated process during which a variety of metabolic abnormalities occur, and the induction of heparanase may be one of them. The protective effect of heparin and bFGF alone or in combination was more evident in heparinase I versus high glucose injury indicating the limited damage induced by heparinase I and the complexity of glucose-induced cell injury. Cell injury by heparinase I further confirms that degradation of HSPG on the EC surface or the ECM contributes to the diabetic vasculopathy consistent with previous observations, both *in vivo *and *in vitro*. Our findings are the first to show the protective effects of heparin and/or insulin and/or bFGF on cells injured by high glucose or heparinase I. Interestingly, we found that the protective effects of bFGF in the presence of heparin or insulin in cell medium were more evident when cells were treated with heparinase I compared to high glucose. Regardless of the interaction between heparin, insulin and bFGF, this study demonstrated that these three compounds in combination protect cells from high glucose or heparanase injury. These findings provide the basis for further studies in the understanding and treatment of diabetic vascular complications.

## List of abbreviations

bFGF-basic fibroblast growth factor

bFGFR-basic fibroblast growth factor receptor

CHO-Chinese Hamster Ovary

CMF-DPBS-calcium- and magnesium-free Dulbecco's phosphate-buffered saline

CTAP-III-connective tissue activating peptide III

ECM-extracellular matrix

EC(s)-endothelial cell(s)

GAG-glycosaminoglycan

GBM-glomerular basement membrane

HIP-HS/heparin-interacting proteinHS- heparan sulfate

HSPG(s)-Heparan sulfate proteoglycan(s)

LDH-lactate dehydrogenase

NO-nitric oxide

PAEC(s)-Porcine Aortic Endothelial Cell(s)

SE-standard error

vWF-von Willebrand Factor

## Competing interests

The author(s) declare that they have no competing interests.

## Authors' contributions

JH participated in the design of the study carried out the experiments and drafted the manuscript. AM conceived the study and participated in the design. LH conceived the study, participated in the design and co-ordination and contributed to the writing of the manuscript.
